# (*S*)-3-[(*S*,*E*)-4-(4-Chloro­phen­yl)-1-nitro­but-3-en-2-yl]thian-4-one

**DOI:** 10.1107/S1600536809043608

**Published:** 2009-10-28

**Authors:** Zhaobo Li, Yifeng Wang, Yi Guo, Shuping Luo

**Affiliations:** a State Key Laboratory Breeding Base of Green Chemistry-Synthesis Technology, Zhejiang University of Technology, Hangzhou 310014, People’s Republic of China

## Abstract

The title compound, C_15_H_16_ClNO_3_S, was obtained by the organocatalytic asymmetric Michael addition of thian-4-one to 1-chloro-4-[(1*E*,3*E*)-4-nitro­buta-1,3-dien­yl]benzene. The double bond has an *E* configuration and the thian-4-one six-membered ring adopts a chair conformation. The crystal structure is stabilized by weak inter­molecular C—H⋯O hydrogen bonds.

## Related literature

For asymmetric Michael addition employing chiral organo­catalysts, see: Belot *et al.* (2008 ▶); Dalko & Moisan (2004 ▶); Xu *et al.* (2008 ▶); Yu *et al.* (2009 ▶).
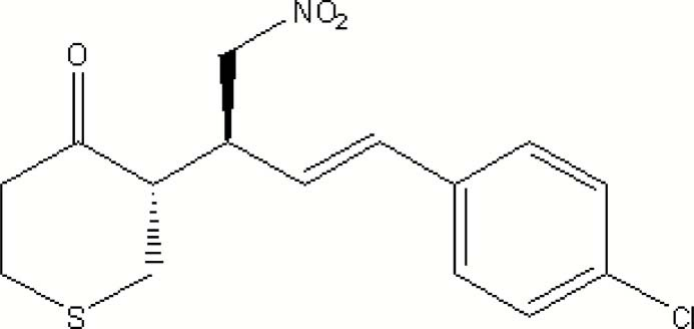


## Experimental

### 

#### Crystal data

C_15_H_16_ClNO_3_S
*M* *_r_* = 325.80
Orthorhombic, 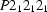

*a* = 5.5220 (2) Å
*b* = 8.3833 (3) Å
*c* = 34.7414 (12) Å
*V* = 1608.27 (10) Å^3^
*Z* = 4
Mo *K*α radiation
μ = 0.38 mm^−1^
*T* = 296 K
0.34 × 0.28 × 0.19 mm

#### Data collection

Rigaku R-AXIS RAPID diffractometer
Absorption correction: multi-scan (*ABSCOR*; Higashi, 1995 ▶) *T*_min_ = 0.865, *T*_max_ = 0.932
15960 measured reflections
3666 independent reflections
2918 reflections with *I* > 2σ(*I*)
*R*_int_ = 0.024

#### Refinement

*R*[*F* ^2^ > 2σ(*F* ^2^)] = 0.032
*wR*(*F* ^2^) = 0.090
*S* = 1.00
3666 reflections
191 parameters
H-atom parameters constrained
Δρ_max_ = 0.16 e Å^−3^
Δρ_min_ = −0.20 e Å^−3^
Absolute structure: Flack (1983 ▶), 1501 Friedel pairs
Flack parameter: 0.03 (7)

### 

Data collection: *PROCESS-AUTO* (Rigaku, 2006 ▶); cell refinement: *PROCESS-AUTO*; data reduction: *CrystalStructure* (Rigaku/MSC, 2007 ▶); program(s) used to solve structure: *SHELXS97* (Sheldrick, 2008 ▶); program(s) used to refine structure: *SHELXL97* (Sheldrick, 2008 ▶); molecular graphics: *ORTEP-3 for Windows* (Farrugia, 1997 ▶); software used to prepare material for publication: *WinGX* (Farrugia, 1999 ▶).

## Supplementary Material

Crystal structure: contains datablocks global, I. DOI: 10.1107/S1600536809043608/fk2005sup1.cif

Structure factors: contains datablocks I. DOI: 10.1107/S1600536809043608/fk2005Isup2.hkl

Additional supplementary materials:  crystallographic information; 3D view; checkCIF report

## Figures and Tables

**Table 1:** Hydrogen-bond geometry (Å, °)

*D*—H⋯*A*	*D*—H	H⋯*A*	*D*⋯*A*	*D*—H⋯*A*
C7—H7*B*⋯O2^i^	0.97	2.45	3.368 (4)	158
C2—H2*B*⋯O1^i^	0.97	2.58	3.484 (3)	156

Symmetry code: (i) 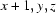.

**Fig. 1.**
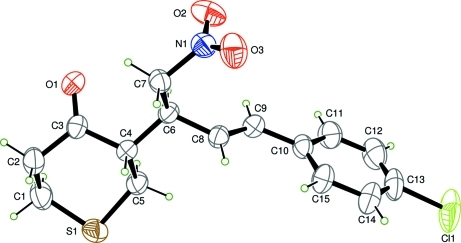
The asymmetric unit of the title compound with the atomic labeling scheme; displacement ellipsoids are drawn at the 50% probability level.

**Fig. 2.**
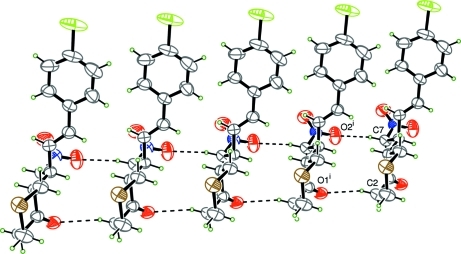
The view of intermolecular interaction illustrated as dash lines.

## supplementary crystallographic information

### Comment

As one of the most important chiral carbon-carbon bond-forming processes in modern organic chemistry, the field of asymmetric Michael addition employing chiral organocatalysts has gained more and more attention and become the focus of intense research efforts (Dalko & Moisan, 2004; Belot *et al.*, 2008; Yu *et al.*, 2009). Consequently, we have synthesized a series of Michael adducts by employing *cyclo*-ketones to nitrodienes in our laboratory. We report here the crystal structure and the absolute configuration of the title compound, (I). The six-membered ring of thian-4-one adopts a chair conformation. The C8═C9 bond involves the *E* configuration with the C6—C8—C9—C10 torsion angle of 178.1 (17)°. The conformation of (I) is stabilized by weak intermolecular C7—H7B···O2 and C2—H2B···O1 interaction, Table 1, Fig 2.

### Experimental

A 1,2-dichloroethane (0.5 ml) solution of thian-4-one (0.25 mmol) and 1-chloro-4-((1*E*,3*E*)-4-nitrobuta-1,3-dienyl)benzene (0.25 mmol) in the presence of (*S*)-1-methyl-2-(pyrrolidin-2-ylmethylthio)-1*H*-imidazole (0.025 mmol) as amine catalyst and (*R*)-2-(3-(3,5-bis(trifluoromethyl)phenyl)thioureido)-2-phenylacetic acid (0.025 mmol) as acid module at room tempreture was stirred vigorously (Xu *et al.*, 2008). After completion of the reaction, the resulted reaction mixture was purified directly by silica gel column chromatography (eluent: petroleum ether-EtOAc). Single crystals were obtained by slow evaporation of an ethanol-EtOAc solution.

### Refinement

All carbon-bonded H atoms were placed in calculated positions with C—H = 0.93 Å (aromatic), C—H = 0.98 Å (*sp^2^*), C—H = 0.97 Å (*sp*^3^) and refined using a riding model, with *U*_iso_(H)=1.2_eq_(C).

### Crystal data

**Table:**

C_15_H_16_ClNO_3_S	*F*(000) = 680
*M**_r_* = 325.80	*D*_x_ = 1.346 Mg m^−^^3^
Orthorhombic, *P*2_1_2_1_2_1_	Mo *K*α radiation, λ = 0.71073 Å
Hall symbol: P 2ac 2ab	Cell parameters from 12670 reflections
*a* = 5.5220 (2) Å	θ = 3.0–27.5°
*b* = 8.3833 (3) Å	µ = 0.38 mm^−^^1^
*c* = 34.7414 (12) Å	*T* = 296 K
*V* = 1608.27 (10) Å^3^	Block, colorless
*Z* = 4	0.34 × 0.28 × 0.19 mm

### Data collection

**Table:**

Rigaku R-AXIS RAPID diffractometer	3666 independent reflections
Radiation source: rotating anode	2918 reflections with *I* > 2σ(*I*)
graphite	*R*_int_ = 0.024
Detector resolution: 10.00 pixels mm^-1^	θ_max_ = 27.5°, θ_min_ = 3.0°
ω scans	*h* = −7→6
Absorption correction: multi-scan (*ABSCOR*; Higashi, 1995)	*k* = −10→10
*T*_min_ = 0.865, *T*_max_ = 0.932	*l* = −45→45
15960 measured reflections	

### Refinement

**Table:**

Refinement on *F*^2^	Hydrogen site location: inferred from neighbouring sites
Least-squares matrix: full	H-atom parameters constrained
*R*[*F*^2^ > 2σ(*F*^2^)] = 0.032	*w* = 1/[σ^2^(*F*_o_^2^) + (0.047*P*)^2^ + 0.182*P*] where *P* = (*F*_o_^2^ + 2*F*_c_^2^)/3
*wR*(*F*^2^) = 0.090	(Δ/σ)_max_ < 0.001
*S* = 1.00	Δρ_max_ = 0.16 e Å^−^^3^
3666 reflections	Δρ_min_ = −0.20 e Å^−^^3^
191 parameters	Extinction correction: *SHELXL97* (Sheldrick, 2008), Fc^*^=kFc[1+0.001xFc^2^λ^3^/sin(2θ)]^-1/4^
0 restraints	Extinction coefficient: 0.0061 (13)
Primary atom site location: structure-invariant direct methods	Absolute structure: Flack (1983), 1501 Friedel pairs
Secondary atom site location: difference Fourier map	Flack parameter: 0.03 (7)

### Special details

**Table:**

Geometry. All e.s.d.'s (except the e.s.d. in the dihedral angle between two l.s. planes) are estimated using the full covariance matrix. The cell e.s.d.'s are taken into account individually in the estimation of e.s.d.'s in distances, angles and torsion angles; correlations between e.s.d.'s in cell parameters are only used when they are defined by crystal symmetry. An approximate (isotropic) treatment of cell e.s.d.'s is used for estimating e.s.d.'s involving l.s. planes.
Refinement. Refinement of *F*^2^ against ALL reflections. The weighted *R*-factor *wR* and goodness of fit *S* are based on *F*^2^, conventional *R*-factors *R* are based on *F*, with *F* set to zero for negative *F*^2^. The threshold expression of *F*^2^ > σ(*F*^2^) is used only for calculating *R*-factors(gt) *etc*. and is not relevant to the choice of reflections for refinement. *R*-factors based on *F*^2^ are statistically about twice as large as those based on *F*, and *R*- factors based on ALL data will be even larger.

### Fractional atomic coordinates and isotropic or equivalent isotropic displacement parameters (Å^2^)

**Table:**

	*x*	*y*	*z*	*U*_iso_*/*U*_eq_	
C13	−0.1203 (6)	0.9836 (3)	0.01706 (6)	0.0747 (7)	
S1	0.55221 (10)	0.17716 (7)	0.117335 (15)	0.06690 (17)	
Cl1	−0.1215 (2)	1.11399 (9)	−0.022097 (18)	0.1245 (4)	
C6	0.0659 (3)	0.5010 (2)	0.16547 (4)	0.0447 (4)	
H6	−0.0877	0.4429	0.1634	0.054*	
O1	0.0671 (3)	0.22754 (16)	0.21132 (4)	0.0613 (3)	
C4	0.2745 (3)	0.3803 (2)	0.16338 (4)	0.0444 (4)	
H4	0.4264	0.4371	0.1684	0.053*	
N1	−0.1196 (3)	0.7113 (2)	0.20576 (4)	0.0555 (4)	
C5	0.2912 (4)	0.3026 (2)	0.12328 (5)	0.0555 (4)	
H5A	0.2954	0.3859	0.1039	0.067*	
H5B	0.1466	0.2394	0.1189	0.067*	
C8	0.0775 (3)	0.6222 (2)	0.13348 (5)	0.0476 (4)	
H8	0.2189	0.6816	0.1310	0.057*	
C7	0.0736 (3)	0.5865 (2)	0.20462 (5)	0.0496 (4)	
H7A	0.0481	0.5101	0.2252	0.059*	
H7B	0.2309	0.6356	0.2083	0.059*	
C3	0.2507 (3)	0.2460 (2)	0.19269 (5)	0.0492 (4)	
C9	−0.0980 (3)	0.6500 (2)	0.10879 (5)	0.0522 (4)	
H9	−0.2357	0.5869	0.1113	0.063*	
O3	−0.0587 (3)	0.84976 (18)	0.20651 (5)	0.0819 (5)	
C2	0.4615 (4)	0.1319 (3)	0.19524 (6)	0.0669 (5)	
H2A	0.4315	0.0550	0.2155	0.080*	
H2B	0.6073	0.1906	0.2017	0.080*	
C15	0.0781 (4)	0.8818 (2)	0.07241 (5)	0.0631 (5)	
H15	0.2076	0.8847	0.0895	0.076*	
O2	−0.3289 (3)	0.6674 (2)	0.20483 (5)	0.0838 (5)	
C10	−0.1020 (3)	0.7694 (2)	0.07758 (5)	0.0519 (4)	
C1	0.4989 (5)	0.0446 (3)	0.15734 (6)	0.0757 (6)	
H1A	0.3568	−0.0196	0.1519	0.091*	
H1B	0.6359	−0.0270	0.1599	0.091*	
C11	−0.2923 (4)	0.7687 (3)	0.05161 (6)	0.0691 (6)	
H11	−0.4169	0.6951	0.0547	0.083*	
C12	−0.3010 (5)	0.8756 (3)	0.02114 (6)	0.0819 (7)	
H12	−0.4291	0.8732	0.0038	0.098*	
C14	0.0704 (5)	0.9901 (3)	0.04238 (6)	0.0745 (6)	
H14	0.1920	1.0658	0.0394	0.089*	

### Atomic displacement parameters (Å^2^)

**Table:**

	*U*^11^	*U*^22^	*U*^33^	*U*^12^	*U*^13^	*U*^23^
C13	0.122 (2)	0.0560 (12)	0.0463 (9)	0.0278 (14)	−0.0102 (12)	0.0075 (8)
S1	0.0674 (3)	0.0707 (3)	0.0626 (3)	0.0147 (3)	0.0095 (2)	−0.0069 (2)
Cl1	0.2253 (11)	0.0834 (4)	0.0646 (3)	0.0384 (6)	−0.0194 (5)	0.0284 (3)
C6	0.0489 (8)	0.0463 (8)	0.0387 (7)	−0.0005 (8)	0.0019 (8)	0.0031 (6)
O1	0.0645 (8)	0.0616 (8)	0.0578 (7)	−0.0018 (7)	0.0091 (7)	0.0157 (6)
C4	0.0466 (8)	0.0457 (9)	0.0410 (8)	−0.0008 (7)	0.0026 (7)	0.0035 (7)
N1	0.0581 (9)	0.0606 (10)	0.0478 (8)	0.0061 (8)	−0.0027 (7)	−0.0059 (7)
C5	0.0667 (10)	0.0568 (11)	0.0428 (8)	0.0090 (9)	0.0022 (8)	0.0012 (8)
C8	0.0539 (10)	0.0429 (8)	0.0460 (8)	0.0007 (8)	0.0025 (8)	0.0031 (7)
C7	0.0516 (9)	0.0514 (10)	0.0457 (8)	0.0085 (8)	−0.0046 (8)	−0.0011 (7)
C3	0.0569 (10)	0.0486 (10)	0.0420 (8)	0.0003 (8)	−0.0041 (8)	0.0029 (7)
C9	0.0555 (10)	0.0536 (10)	0.0475 (9)	−0.0008 (9)	0.0003 (8)	0.0036 (7)
O3	0.1031 (12)	0.0509 (9)	0.0918 (11)	0.0066 (9)	−0.0103 (10)	−0.0046 (8)
C2	0.0719 (12)	0.0675 (13)	0.0615 (11)	0.0137 (11)	−0.0082 (10)	0.0124 (9)
C15	0.0780 (13)	0.0597 (11)	0.0516 (10)	−0.0048 (11)	−0.0136 (10)	0.0107 (8)
O2	0.0491 (8)	0.1071 (13)	0.0954 (11)	0.0053 (9)	0.0029 (7)	−0.0237 (11)
C10	0.0614 (10)	0.0519 (10)	0.0423 (8)	0.0094 (9)	−0.0049 (8)	0.0009 (7)
C1	0.0906 (16)	0.0619 (13)	0.0746 (13)	0.0252 (12)	−0.0040 (12)	0.0032 (10)
C11	0.0678 (12)	0.0778 (15)	0.0618 (11)	0.0038 (11)	−0.0150 (10)	0.0045 (10)
C12	0.0969 (18)	0.0897 (18)	0.0590 (11)	0.0216 (16)	−0.0267 (12)	0.0023 (12)
C14	0.1063 (17)	0.0566 (11)	0.0606 (11)	−0.0037 (14)	−0.0062 (13)	0.0150 (9)

### Geometric parameters (Å, °)

**Table:**

C13—C12	1.355 (4)	C8—H8	0.9300
C13—C14	1.373 (4)	C7—H7A	0.9700
C13—Cl1	1.745 (2)	C7—H7B	0.9700
S1—C5	1.7959 (19)	C3—C2	1.509 (3)
S1—C1	1.804 (2)	C9—C10	1.476 (2)
C6—C8	1.507 (2)	C9—H9	0.9300
C6—C4	1.534 (2)	C2—C1	1.520 (3)
C6—C7	1.539 (2)	C2—H2A	0.9700
C6—H6	0.9800	C2—H2B	0.9700
O1—C3	1.213 (2)	C15—C10	1.382 (3)
C4—C3	1.524 (2)	C15—C14	1.383 (3)
C4—C5	1.541 (2)	C15—H15	0.9300
C4—H4	0.9800	C10—C11	1.385 (3)
N1—O3	1.209 (2)	C1—H1A	0.9700
N1—O2	1.213 (2)	C1—H1B	0.9700
N1—C7	1.494 (2)	C11—C12	1.388 (3)
C5—H5A	0.9700	C11—H11	0.9300
C5—H5B	0.9700	C12—H12	0.9300
C8—C9	1.315 (3)	C14—H14	0.9300
			
C12—C13—C14	121.62 (19)	O1—C3—C2	122.19 (16)
C12—C13—Cl1	119.81 (19)	O1—C3—C4	121.53 (16)
C14—C13—Cl1	118.5 (2)	C2—C3—C4	116.17 (15)
C5—S1—C1	98.11 (10)	C8—C9—C10	127.61 (17)
C8—C6—C4	112.19 (13)	C8—C9—H9	116.2
C8—C6—C7	109.65 (14)	C10—C9—H9	116.2
C4—C6—C7	109.17 (13)	C3—C2—C1	111.04 (16)
C8—C6—H6	108.6	C3—C2—H2A	109.4
C4—C6—H6	108.6	C1—C2—H2A	109.4
C7—C6—H6	108.6	C3—C2—H2B	109.4
C3—C4—C6	113.01 (13)	C1—C2—H2B	109.4
C3—C4—C5	107.25 (14)	H2A—C2—H2B	108.0
C6—C4—C5	111.50 (13)	C10—C15—C14	121.53 (19)
C3—C4—H4	108.3	C10—C15—H15	119.2
C6—C4—H4	108.3	C14—C15—H15	119.2
C5—C4—H4	108.3	C11—C10—C15	117.69 (18)
O3—N1—O2	123.82 (19)	C11—C10—C9	119.13 (18)
O3—N1—C7	118.28 (17)	C15—C10—C9	123.17 (16)
O2—N1—C7	117.86 (18)	C2—C1—S1	113.15 (17)
C4—C5—S1	113.56 (12)	C2—C1—H1A	108.9
C4—C5—H5A	108.9	S1—C1—H1A	108.9
S1—C5—H5A	108.9	C2—C1—H1B	108.9
C4—C5—H5B	108.9	S1—C1—H1B	108.9
S1—C5—H5B	108.9	H1A—C1—H1B	107.8
H5A—C5—H5B	107.7	C10—C11—C12	121.3 (2)
C9—C8—C6	124.67 (17)	C10—C11—H11	119.3
C9—C8—H8	117.7	C12—C11—H11	119.3
C6—C8—H8	117.7	C13—C12—C11	119.1 (2)
N1—C7—C6	109.27 (13)	C13—C12—H12	120.5
N1—C7—H7A	109.8	C11—C12—H12	120.5
C6—C7—H7A	109.8	C13—C14—C15	118.7 (2)
N1—C7—H7B	109.8	C13—C14—H14	120.6
C6—C7—H7B	109.8	C15—C14—H14	120.6
H7A—C7—H7B	108.3		
			
C8—C6—C4—C3	−173.58 (14)	C6—C8—C9—C10	178.10 (17)
C7—C6—C4—C3	64.67 (18)	O1—C3—C2—C1	114.2 (2)
C8—C6—C4—C5	−52.68 (19)	C4—C3—C2—C1	−62.0 (2)
C7—C6—C4—C5	−174.43 (14)	C14—C15—C10—C11	−0.1 (3)
C3—C4—C5—S1	−62.42 (17)	C14—C15—C10—C9	178.8 (2)
C6—C4—C5—S1	173.37 (12)	C8—C9—C10—C11	172.61 (19)
C1—S1—C5—C4	56.38 (16)	C8—C9—C10—C15	−6.3 (3)
C4—C6—C8—C9	123.85 (19)	C3—C2—C1—S1	58.3 (2)
C7—C6—C8—C9	−114.67 (19)	C5—S1—C1—C2	−53.16 (18)
O3—N1—C7—C6	−112.49 (18)	C15—C10—C11—C12	0.8 (3)
O2—N1—C7—C6	65.5 (2)	C9—C10—C11—C12	−178.2 (2)
C8—C6—C7—N1	53.18 (19)	C14—C13—C12—C11	−0.2 (4)
C4—C6—C7—N1	176.45 (14)	Cl1—C13—C12—C11	177.53 (19)
C6—C4—C3—O1	9.8 (2)	C10—C11—C12—C13	−0.6 (4)
C5—C4—C3—O1	−113.44 (18)	C12—C13—C14—C15	0.9 (4)
C6—C4—C3—C2	−173.94 (15)	Cl1—C13—C14—C15	−176.91 (18)
C5—C4—C3—C2	62.78 (19)	C10—C15—C14—C13	−0.7 (3)

### Hydrogen-bond geometry (Å, °)

**Table:**

*D*—H···*A*	*D*—H	H···*A*	*D*···*A*	*D*—H···*A*
C7—H7B···O2^i^	0.97	2.45	3.368 (4)	158
C2—H2B···O1^i^	0.97	2.58	3.484 (3)	156

Symmetry codes: (i) *x*+1, *y*, *z*.
